# Self-reported exposure to intimate partner violence among women and men in Sweden: results from a population-based survey

**DOI:** 10.1186/1471-2458-13-845

**Published:** 2013-09-13

**Authors:** Lotta Nybergh, Charles Taft, Viveka Enander, Gunilla Krantz

**Affiliations:** 1 Department of Public Health and Community Medicine, Institute of Medicine, The Sahlgrenska Academy at University of Gothenburg, Arvid Wallgrens Backe 7, PO Box 453, SE-405 30 Gothenburg, Sweden; 2 The Västra Götaland Region Competence Centre on Intimate Partner Violence, Kungsgatan 12, floor 6, 411 19 Gothenburg, Sweden; 3 Institute of Health and Care Sciences, The University of Gothenburg Centre for Person-centred Care, The Sahlgrenska Academy at University of Gothenburg, Arvid Wallgrens Backe, PO Box 457, SE-405 30 Gothenburg, Sweden; 4 Department of Social Work, University of Gothenburg, Sprängkullsgatan 23, PO Box 720, SE-405 30 Gothenburg, Sweden

**Keywords:** Intimate partner violence, Sweden, WHO VAW instrument, Men, Women

## Abstract

**Background:**

Few population-based studies assessing IPV among randomly selected women and men have been conducted in Sweden. Hence, the aim of the current study was to explore self-reported exposure, associated factors, social and behavioural consequences of and reasons given for using psychological, physical and sexual intimate partner violence (IPV) among women and men residing in Sweden.

**Methods:**

Cross-sectional postal survey of women and men aged 18–65 years. Bivariate and multivariate logistic regression analyses were used to identify factors associated with exposure to IPV.

**Results:**

Past-year IPV exposure rates were similar in women and men; however, earlier-in-life estimates were higher in women. Poor to moderate social support, growing up with domestic violence and being single, widowed or divorced were associated with exposure to all forms of IPV in men and women. Women and men tended to report different social consequences of IPV.

**Conclusions:**

Our finding that women reported greater exposure to IPV earlier-in-life but not during the past year suggests the importance of taking this time frame into account when assessing gender differences in IPV. In-depth, qualitative studies that consider masculinities, femininities power and gender orders would be beneficial for extending and deepening our understanding of the gendered matter of IPV.

## Background

After continuous efforts from women’s rights movements worldwide, violence against women has been recognized as a human rights violation [1] and public health problem of global magnitude [2]. There is now a considerable body of research on men’s violence against female partners worldwide, as well as on its health consequences and associated factors [3]. Recently, men’s victimization of intimate partner violence (IPV) has gained growing attention especially in high-income countries and there are a burgeoning number of studies assessing heterosexual IPV exposure among men [4-6].

Some studies that have found similar levels of either exposure to or use of IPV among women and men have questioned the centrality and relevance of gender to the analysis of partner violence [7]. However, scholars have pointed out that even when the levels of violence may be similar, differences are found in the consequences, meaning, impact, context and ways in which gender is performed within a violent heterosexual relationship [8]. As such, the conceptualization of “gender” can be extended beyond the sex-difference of a particular violent act, to something that is actively “done” in everyday life in a way that shapes, confirms or challenges societal norms of masculinities and femininities. These norms, in turn, are informed by gender hierarchies and power [9] that may influence the experience, consequences and context in which IPV takes place [10,11].

Indeed the focus of most IPV research today has moved beyond considering the simple frequency of a violent act, and often considers other aspects of IPV, such as its severity, patterns, meaning, consequences and motivations [12]. Such studies show, for example, that men belittle women’s use of physical violence against them and react to IPV with laughter and ridicule, [8,13] while women report greater fear of their partner [14] and feel less able to stop physically violent events than men [15]. Researchers have suggested that women’s use of violence should be understood in the larger context of their ongoing victimization, [16] whereas men’s use of violence often occurs in the context of coercive control [17]. Although contradictory research findings exist, [18] it is generally found that women experience more adverse physical [19] and mental [20] consequences, endure longer lasting violence, [21] more severe violence, [22] more coercive control, [5] more overlapping forms of violence, [4] stalking, [15] are less satisfied with their violent relationships and more likely to be victims of sexual violence than men [4,5]. Nonetheless, men who report IPV victimization have significantly poorer health and experience a range of negative psychosocial outcomes compared to men who do not report IPV [23]. Psychological violence is more rarely assessed than physical and sexual IPV in quantitative studies, and its definitions vary considerably. However, some studies find that it is more prevalent than physical or sexual IPV and that exposure rates are similar for men and women [4,24,25]. It has also been suggested that studies which find similar levels of IPV among women and men rely largely on past-year estimates or current partners, whereas studies assessing a longer time-frame or including previous partners tend to find dissimilar levels of violence [11]. Finally, it is noteworthy that important differences in IPV exposure exist within gender groups as well in relation to age, sexuality, ethnicity, (dis)abilities and other social categories [26].

Although many studies on IPV have been conducted in other countries, only one previous population-based study assessing IPV among randomly selected women and men has been conducted in Sweden [6]. That study used the Conflict Tactics Scale whereas the present study uses items from the World Health Organization’s (WHO) Violence Against Women instrument (VAWI). The aim of the current study was to assess IPV exposure among adult women and men residing in Sweden. The specific aims were to explore self-reported 1) exposure to psychological, physical and sexual violence with regard to co-occurrence, prevalence and frequency during the past year and earlier in life by use of the VAWI; 2) socio-demographic and psychosocial factors associated with IPV; 3) social and behavioural consequences of IPV; 4) own use of violence and reasons given for using such violence by women and men.

## Methods

### Sample

A postal questionnaire was sent out through Statistics Sweden between January and March 2009 to a random national sample of 1006 women and 1009 men aged 18–65 years and residing in Sweden. A total of 624 women (62.0%) and 458 men (45.5%) returned the questionnaire. Respondents with missing values on all the violence items (8.2% women and 12.9% men) were excluded from the analyses, resulting in a total sample of 573 women and 399 men. The questionnaire included items on exposure to psychological, physical and sexual IPV, socio-demographic and psychosocial factors, own use of violence, reasons given for using violence and social and behavioural consequences of being exposed to violence.

### Comparisons between respondents and non-respondents

Statistically significant differences between non-respondents and the final sample were assessed by using a two-proportion z-test with a Bonferroni adjustment to the alpha level. Variables included in the analysis were age, country of birth, civil status and individual annual income before tax.

A significantly larger proportion of the non-respondents (n = 382 women and 551 men) were 18–29 years old, unmarried, foreign born and had a low annual income (0–159,999 Swedish crowns). A similar pattern was also found among those with missing values on all violence items (n = 51 women and 55 men), i.e. they were 18–29 years old, unmarried and had a low annual income as defined above.

### Measures

#### ***Intimate partner violence***

The WHO’s Violence Against Women instrument (VAWI) assessing psychological (4 items), physical (6 items) and sexual (3 items) violence committed by an intimate partner was used [27]. The VAWI is, to the best of our knowledge, the only IPV instrument whose psychometric properties have been explored in a Swedish context among both women and men [28,29]. These studies were conducted on the same female and male samples as used in the current study. Support for the VAWI’s construct validity and internal reliability among women has also been found by studies conducted in other countries [30-32]. For each question respondents were asked how often they had experienced a specific act during the past twelve months. Response options were 0 times, 1 time, 2 times, 3–5 times or > 5 times. The response options of 1 and 2 times were combined into a single category. Respondents were also asked if they had experienced the violent act earlier in life.

#### ***Dependent variables***

Psychological violence and a combined variable for physical and sexual violence (henceforth physical/sexual violence) were used as dichotomous outcome variables (unexposed vs. exposed to at least one act of violence) in the bivariate and multivariate logistic regression analyses. In order to increase statistical power, the past-year and earlier-in-life variables were merged into dichotomous life-time variables.

#### ***Independent variables***

A range of socio-demographic and psychosocial variables were investigated. Social support was assessed by asking “At times one needs help and support from someone. Do you have a relative or friend who will help you when…”, followed by four different situations where help and support might be needed: “…you get sick”, “…you need company”, “…you need to speak to someone about personal concerns” and “…you need a loan over 15,000 Swedish crowns”. An answer in the affirmative to all of the questions was coded good social support, whereas answering “no” or “unsure” to any of the questions was considered poor to moderate social support.

#### ***Social and behavioural consequences of violence to everyday life, own use of violence and reasons for using such violence***

Respondents were asked whether they, as a consequence of having been exposed to IPV, had needed to make changes to their everyday lives in order to protect themselves. Furthermore, they were asked if they had used violence against their partner (yes/no), and if the respondent answered affirmatively, further questions inquired about which type of violence it was (psychological, physical or sexual) and reasons for using violence. Due to the exploratory nature of this study, a variety of closed questions followed by an open option for the consequences of violence and own use of violence were used. Results from the most frequently reported answers are given.

### Statistical analyses

The Predictive Analytics SoftWare (PASW) statistical package version 19 and 20 were used and all analyses were conducted separately for women and men.

Differences in socio-demographic factors between women and men were checked for by the Chi-squared test for independence for all categorical variables (civil status, employment status, country of birth, access to social support and having grown up in a home with violence). The Mann Whitney U test was used for the remaining ordinal variables. Differences between women’s and men’s responses to consequences of IPV and reasons for own use of violence were analyzed using the z-test for proportions. Fisher’s exact probability test was used when a cell had less than 5 cases (p-value < 0.05).

Bivariate analyses were performed between socio-demographic and psychosocial factors and exposure to lifetime psychological and physical/sexual violence. The analyses were repeated with dichotomized variables in order to increase statistical power for the multivariate analyses (not in Table). Statistically significant, dichotomized factors at the 0.05 significance level were included simultaneously in a multivariate logistic regression analysis to obtain adjusted odds ratios (OR) of the associations. Once a final model was obtained, those variables that had not met the inclusion criteria based on statistical significance were entered into the final model one at a time to see if they would contribute significantly to the model. Finally, theoretically relevant interaction terms were tested for significance. As duration of the present relationship and civil status correlated above 0.40 for women (r = .42) and men (r = .50), as did duration of the present relationship and age for men (r = .55), duration of the present relationship was excluded from the multivariate analyses. Further multicollinearity could not be detected as the Tolerance value was above .40 and the Variance Inflation Factor was below 2.5 for all variables.

The co-occurrence of psychological, physical and sexual IPV was illustrated by Venn diagrams separately for the past-year and earlier-in-life time frames.

### Ethical considerations

The study conformed with the WHO ethical and safety recommendations for research on domestic violence against women [33] and received ethical approval from the Regional Ethics Review Board in Gothenburg (Dnr: 527–08). Only one eligible person per household was chosen for ethical and safety reasons. Full anonymity and confidentiality were guaranteed and contact information for a general practitioner (GK), a psychologist and a contact person at Statistics Sweden was provided for additional information and/or referral.

## Results

### Sample

In comparison to women, men were older, had a lower level of education, were more often employed and had a higher annual income (p < 0.05; Table 1). The majority of the sample was currently in a relationship (85.1% women; 87.9% men) that was heterosexual (98.8% women; 98.7% men) and had lasted over 10 years (59.4% women; 64.3% men; Table 1).

**Table 1:** **Socio**-**demographic and psychosocial factors of the total sample**, **men and women**

	**Men N = 399**		**Women N = 573**		P-value*
**Characteristic**	**%**	**(N)**	**%**	**(N)**	
Age groups					
18–29	14.3	(57)	18.7	(107)	.007
30–39	19.3	(77)	24.1	(138)	
40–49	24.1	(96)	21.8	(125)	
50–59	24.6	(98)	23.7	(136)	
60–65	17.8	(71)	11.7	(67)	
Civil status					
Single/widowed/divorced	12.1	(48)	14.9	(85)	.325
Boyfriend/girlfriend	13.4	(53)	11.2	(64)	
Married/cohabitant/registered partnership	74.6	(296)	73.8	(420)	
Heterosexual relationship	86.2	(344)	83.2	(477)	
Same-sex relationship	1.3	(5)	1.2	(7)	
Duration of the present relationship					
> 10 years	56.9	(227)	50.3	(288)	.298
4–10 years	15.5	(62)	20.1	(115)	
1–3 years	16.0	(64)	14.3	(82)	
Country of Birth					
Sweden	89.2	(356)	90.6	(519)	.164
Other Nordic country	1.8	(7)	2.6	(15)	
Other European country	2.5	(10)	3.1	(18)	
Country outside of Europe	6.5	(26)	3.7	(21)	
Educational level (highest)					
University	39.4	(156)	47.2	(270)	.047
High school (10–12 yrs)	43.7	(173)	36.9	(211)	
Compulsory (≤9 yrs)	16.9	(67)	15.9	(91)	
Annual income (before tax, Swedish crowns)					
310,000 or more	40.9	(163)	15.2	(87)	.001
235,000–309,999	26.8	(107)	25.0	(143)	
160,000–234,999	13.0	(52)	30.5	(175)	
0–159,999	19.3	(77)	29.3	(168)	
Employment status					
Employed	83.3	(329)	69.7	(396)	<0.0001
Student	5.1	(20)	6.2	(35)	
Retired	5.8	(23)	8.3	(47)	
Sick leave (more than 3 months)	1.3	(5)	1.4	(8)	
Parental leave or leave of absence	0.5	(2)	6.2	(35)	
Unemployed	2.8	(11)	4.0	(23)	
Other	1.3	(5)	4.2	(24)	
Access to social support					
Yes	64.4	(257)	64.6	(370)	.973
No	11.8	(47)	10.5	(60)	
Unsure	23.1	(92)	24.3	(139)	
Grown up in a home with violence between parents					
No	90.4	(357)	90.3	(510)	.953
Yes	9.4	(37)	9.2	(52)	
Unsure	0.3	(1)	0.5	(3)	

*Mann Whitney U test for ordinal variables and Chi-squared test for independence for categorical variables.

### Prevalence, frequency and co-occurrence of psychological, physical and sexual violence

IPV exposure rates during the past year were similar for women and men for all three forms of violence (Table 2). For example, 8.1% (95% CI 5.9–10.3) of the women and 7.6% (95% CI 5.0–10.2) of the men reported physical IPV. For earlier in life, women had higher exposure rates than men for all three forms of violence: psychological IPV was experienced by 23.6% (95% CI 20.1–27.1) of the women and 13.8% (95% CI 10.4–17.2) of the men; physical IPV by 14.3% (95% CI 11.4–17.2) of the women and 6.8% (95% CI 4.3–9.3) of the men; and sexual IPV by 9.2% (95% CI 6.8–11.6) of the women and 2.5% (95% CI 1.0–4.0) of the men. Most respondents were exposed to the first and comparatively less severe IPV item in each sub-scale and the frequency of exposure was generally 1–2 times during the past year (Table 2).

**Table 2:** Violence acts perpetrated by an intimate partner: past year (frequency) and earlier in life (yes/no)

	**Men: N = 399**					**Women: N = 573**				
		**Number of events, past year % ª (N)**					**Number of events, past year % ª (N)**			
**Psychological violence**	**Past year**	**1–2**	**3–5**	**>5**	**Earlier in life**	**Past year**	**1–2**	**3–5**	**>5**	**Earlier in life**
Insulted me in a way that made me feel bad	**20.5 (79)**	13.2 (51)	2.1 (8)	5.2 (20)	12.0 (48)	**20.7 (109)**	13.5 (71)	2.7 (14)	4.6 (24)	19.9 (114)
Belittled and humiliated me in front of others	**9.5 (37)**	6.4 (25)	1.3 (5)	1.8 (7)	6.5 (26)	**7.7 (41)**	5.8 (31)	0.8 (4)	1.1 (6)	13.1 (75)
Tried to scare and terrorize me on purpose	**8.7 (34)**	6.7 (26)	0.3 (1)	1.8 (7)	6.5 (26)	**6.0 (32)**	3.6 (19)	1.5 (8)	0.9 (5)	10.5 (60)
Threatened to hurt me or someone I care about	**1.0 (4)**	0.3 (1)	0.3 (1)	0.5 (2)	2.8 (11)	**1.5 (8)**	0.7 (4)	0.4 (2)	0.4 (2)	7.3 (42)
*Individuals exposed to acts of psychological violence*	*24*.*0* (*92*)				*13*.*8* (*55*)	*23*.*6* (*123*)				*23*.*6* (*135*)
**Physical violence**	**Past year**	**1–2**	**3–5**	**>5**	**Earlier in life**	**Past year**	**1–2**	**3–5**	**>5**	**Earlier in life**
Pushed or shoved me	**5.9 (23)**	4.6 (18)	0.3 (1)	1.0 (4)	5.3 (21)	**7.1 (38)**	5.5 (29)	0.9 (5)	0.8 (4)	2.6 (72)
Thrown something that could have hurt me	**2.8 (11)**	2.3 (9)	0.3 (1)	0.3 (1)	3.3 (13)	**1.7 (9)**	1.3 (7)	0.2 (1)	0.2 (1)	5.1 (29)
Hit me with his/her fist or with some other object	**2.1 (8)**	1.5 (6)	0.3 (1)	0.3 (1)	4.0 (16)	**1.3 (7)**	0.7 (4)	-	0.6 (3)	5.8 (33)
Kicked and dragged me and beaten me up	**0.8 (3)**	0.3 (1)	-	0.5 (2)	1.5 (6)	**0.6 (3)**	0.2 (1)	-	0.4 (2)	3.5 (20)
Choked me or burnt me on purpose	**0.5 (2)**	0.5 (2)	-	-	1.5 (6)	**0.6 (3)**	0.2 (1)	0.4 (2)	-	4.7 (27)
Hurt me with a knife, a gun or some other weapon	-	-	-	-	1.5 (6)	**0.2 (1)**	0.2 (1)	-	-	1.2 (7)
*Individuals exposed to acts of physical violence*	*7*.*6* (*29*)				*6*.*8* (*27*)	*8*.*1* (*43*)				*14*.*3* (*82*)
**Sexual violence**	**Past year**	**1–2**	**3–5**	**>5**	**Earlier in life**	**Past year**	**1–2**	**3–5**	**>5**	**Earlier in life**
Demanded to have sex with me even though I did not want to	**2.3 (9)**	1.8 (7)	-	0.5 (2)	2.3 (9)	**2.6 (14)**	1.5 (8)	0.4 (2)	0.7 (4)	8.2 (47)
Forced me to have sex against my will by using his/her physical strength	**0.3 (1)**	0.3 (1)	-	-	1.3 (5)	**0.4 (2)**	0.2 (1)	-	0.2 (1)	2.6 (15)
Forced me to perform sexual acts that I experienced as degrading and/or humiliating	**0.3 (1)**	0.3 (1)	-	-	1.8 (7)	**0.4 (2)**	0.4 (2)	-	-	4.2 (24)
*Individuals exposed to acts of sexual violence*	*2*.*3* (*9*)				*2*.*5* (*10*)	*3*.*0* (*16*)				*9*.*2* (*53*)
***Summary measures all forms of violence***	***25.6 *( *102 *)**				***15.3 *( *61 *)**	***23.2 *( *133 *)**				***26.0 *( *149 *)**

^ª^Percentage is given in valid percent.

**Figure 1:**
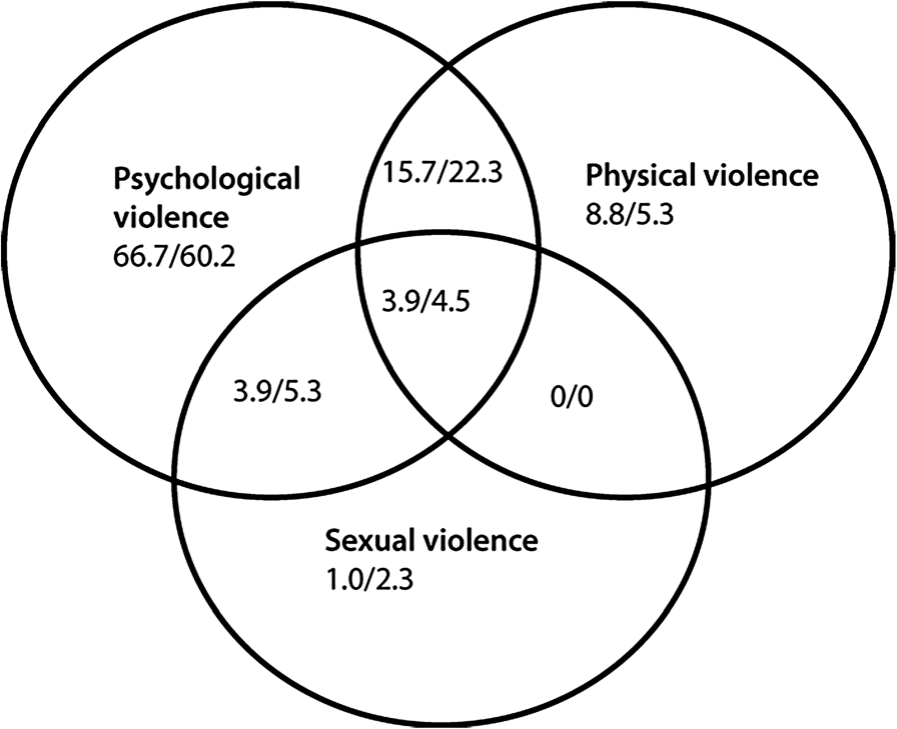
Co-occurrence of psychological, physical and sexual violence among men (N = 102) / women (N = 133) reported for the past-year, given in %.

**Figure 2:**
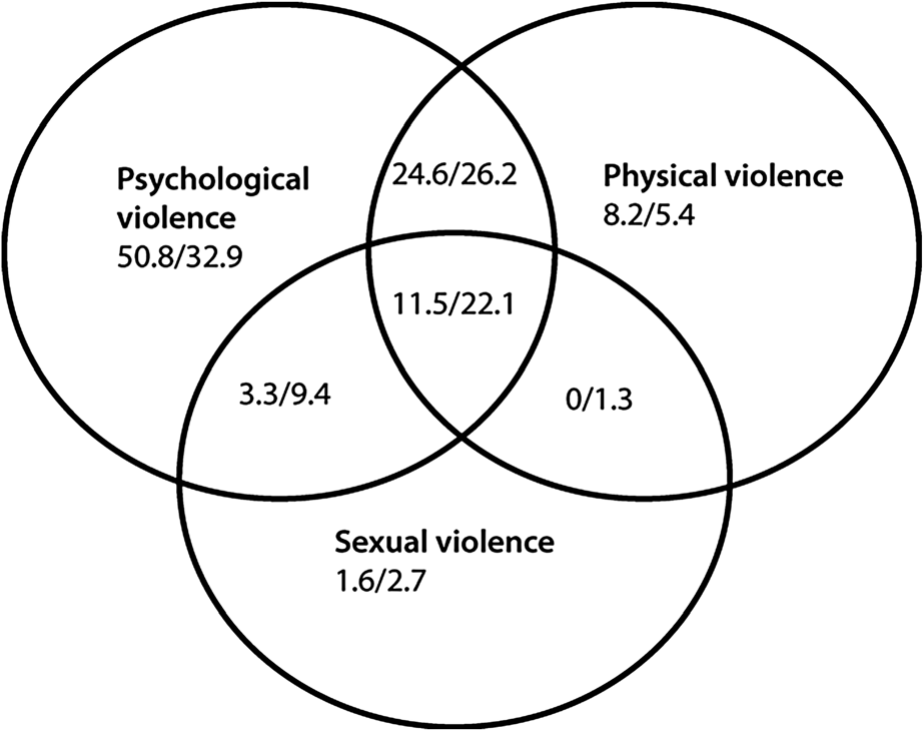
Co-occurrence of psychological, physical and sexual violence among men (N = 61) / women (N = 149) reported for earlier-in-life, given in %.

For both time frames, psychological violence alone was the most frequent form of violence, followed by a co-occurrence with physical violence (Figures 1 and 2). Although the patterns of co-occurrence were similar for women and men during the past year, differences were observed for the earlier-in-life time frame. For example, a greater proportion of women than men were simultaneously exposed to all three forms of IPV.

### Associated socio-demographic and psychosocial factors

Factors associated with psychological IPV in the bivariate analyses were age, civil status, education, duration of the present relationship, social support and having grown up in a home with violence for both women and men (Table 3). Physical/sexual IPV was associated with civil status, duration of present relationship, social support and having grown up in a home with violence for both women and men. Moreover, age, income and partner’s country of birth were associated with physical/sexual IPV for women (Table 4).

**Table 3:** **Associations between psychological violence** (**life**-**time**), **socio**-**demographic and psychosocial factors**

	**Psychological violence**					
	**Men: N = 399 (Exposed N = 123)**			**Women: N = 573 (Exposed N = 212)**		
	**Men (N)**	**Exposed % (N)**	**Crude OR (95% CI)**	**Women (N)**	**Exposed N (%)**	**Crude OR (95% CI)**
**Age groups**						
60–65	71	13.8 (17)	1	67	8.5 (18)	1
50–59	98	20.3 (25)	1.12 (0.55–2.28)	136	19.8 (42)	1.21 (0.63–2.32)
40–49	96	25.2 (31)	1.52 (0.76–3.03)	125	24.1 (51)	1.85 (0.97–3.55)
30–39	77	20.3 (25)	1.53 (0.74–3.15)	138	27.4 (58)	1.92 (1.01–3.64)
18–29	57	20.3 (25)	2.56 (1.20–3.42)	107	20.3 (43)	1.75 (0.90–3.42)
**Civil Status**						
Married/cohabitant/registered partnership	296	59.3 (73)	1	420	60.5 (127)	1
Boyfriend/girlfriend	53	17.1 (21)	2.05 (1.11–3.79)	64	12.9 (27)	1.70 (0.99–2.91)
Single/widowed/divorced	48	23.6 (29)	4.62 (2.45–8.73)	85	26.7 (56)	4.52 (2.74–7.45)
**Duration of Present Relationship**						
>10 yrs	227	45.9 (45)	1	288	48.1 (76)	1
4–10 yrs	62	27.6 (27)	3.09 (1.70–5.62)	115	27.2 (43)	1.64 (1.04–2.60)
≤ 3 yrs	34	26.5 (26)	2.81 (1.55–5.11)	82	24.7 (39)	2.46 (1.48–4.08)
**Country of birth**						
Sweden	356	90.2 (110)	1	517	91.5 (193)	1
Outside Sweden	43	9.8 (12)	0.99 (0.50–1.99)	54	8.5 (18)	0.89 (0.50–1.61)
**Partner**’**s country of birth**						
Sweden	349	84.6 (104)	1	494	83.5 (177)	1
Outside Sweden	50	15.4 (19)	1.43 (0.77–2.64)	79	16.5 (35)	1.43 (0.88–2.31)
**Educational level**						
University	156	46.3 (56)	1	270	49.5 (105)	1
High school (10–12 yrs)	173	43.0 (52)	0.64 (0.39–1.03)	211	38.7 (82)	0.96 (0.67–1.40)
Compulsory (≤9 yrs)	67	10.7 (13)	0.29 (0.13–0.61)	91	11.8 (25)	0.589 (0.348–0.997)
**Annual Income (SEK)**						
310,000 or more	163	39.8 (49)	1	87	16.0 (34)	1
235,000–309,999	107	26.0 (32)	0.98 (0.58–1.68)	143	25.0 (53)	0.83 (0.48–1.45)
160,000–234,999	52	13.8 (17)	1.12 (0.57–2.19)	175	29.2 (62)	0.76 (0.44–1.30)
0–159,999	77	20.3 (25)	1.15 (0.64–2.07)	168	29.7 (63)	0.83 (0.48–1.42)
**Employment status**						
Employed	329	81.0 (98)	1	396	68.2 (144)	1
Student, retired, sick-leave, parental leave or leave of absence, unemployed, other	66	19.0 (23)	1.24 (0.71–2.18)	172	31.8 (67)	1.11 (0.77–1.61)
**Partner**’**s employment status**						
Employed	262	64.5 (71)	1	414	77.1 (138)	1
Student, retired, sick-leave, parental leave or leave of absence, unemployed, other	121	35.5 (39)	1.28 (0.80–2.05)	111	22.9 (41)	1.14 (0.74–1.77)
**Children living at home**						
No	206	52.5 (64)	1	297	50.0 (150)	1
Yes	190	47.5 (58)	0.97 (0.63–1.47)	270	50.0 (105)	1.17 (0.83–1.65)
**Access to social support**						
Good	251	53.3 (65)	1	361	53.6 (111)	1
Poor/moderate	139	46.7 (57)	1.97 (1.27–3.06)	199	46.4 (96)	2.06 (1.44–2.95)
**Grown up in a home with violence**						
No	357	83.5 (101)	1	510	82.5 (170)	1
Yes/Unsure	38	16.5 (20)	2.78 (1.41–5.48)	55	17.5 (36)	3.92 (2.16–7.10)

Total sample n = 972.

**Table 4:** **Associations between physical**/**sexual violence** (**life**-**time**), **socio**-**demographic and psychosocial factors**

	**Physical/sexual violence**					
	**Men: N = 399 (Exposed N = 56)**			**Women: N = 573 (Exposed N = 137)**		
	**Men (N)**	**Exposed % (N)**	**Crude OR (95% CI)**	**Women (N)**	**Exposed % (N)**	**Crude OR (95% CI)**
**Age groups**						
60–65	71	16.1 (9)	1	67	5.8 (8)	1
50–59	98	14.3 (8)	0.62 (0.23–1.69)	136	17.5 (24)	1.63 (0.69–3.86)
40–49	96	30.4 (17)	1.52 (0.63–3.64)	125	23.4 (32)	2.48 (1.07–5.75)
30–39	77	14.3 (8)	0.81 (0.29–2.23)	138	29.2 (40)	2.94 (1.29–6.72)
18–29	57	25.0 (14)	2.26 (0.90–5.70)	107	24.1 (33)	3.22 (1.38–7.51)
**Civil Status**						
Married/cohabitant/registered partnership	296	58.9 (33)	1	420	56.6 (77)	1
Boyfriend/girlfriend	53	14.3 (8)	1.43 (0.62–3.29)	64	15.4 (21)	2.28 (1.27–4.07)
Single/widowed/divorced	48	26.8 (15)	4.06 (1.97–8.35)	85	27.9 (38)	4.44 (2.66–7.42)
**Duration of Present Relationship**						
>10 yrs	227	48.8 (21)	1	288	35.3 (36)	1
4–10 yrs	62	23.3 (10)	1.91 (0.85–4.30)	115	31.4 (32)	2.71 (1.58–4.64)
≤ 3 yrs	64	27.9 (12)	2.33 (1.08–5.05)	82	33.3 (34)	4.92 (2.81–8.62)
**Country of birth**						
Sweden	356	83.6 (46)	1	517	88.3 (121)	1
Outside Sweden	43	16.4 (9)	187 (0.84–4.18)	54	11.7 (16)	1.34 (0.72–2.49)
**Partner**’**s country of birth**						
Sweden	349	89.3 (50)	1	494	79.6 (109)	1
Outside Sweden	50	10.7 (6)	0.88 (0.36–2.19)	79	20.4 (28)	1.92 (1.15–3.20)
**Educational level**						
University	156	48.1 (26)	1	270	48.2 (66)	1
High school (10–12 yrs)	173	40.7 (22)	1.48 (0.57–3.82)	211	38.7 (53)	1.37 (0.75–2.50)
Compulsory (≤9 yrs)	67	11.1 (6)	2.10 (0.82–5.37)	91	13.1 (18)	1.31 (0.73–2.35)
**Annual Income** (**SEK**)						
310,000 or more	163	32.1 (18)	1	87	8.8 (12)	1
235,000–309,999	107	32.1 (18)	1.58 (0.78–3.21)	143	26.3 (36)	2.08 (1.01–4.26)
160,000–234,999	52	8.9 (5)	0.89 (0.31–2.54)	175	29.2 (40)	1.82 (0.90–3.68)
0–159,999	77	26.8 (15)	1.96 (0.93–4.14)	168	35.8 (49)	2.53 (1.26–5.09)
**Employment status**						
Employed	329	80.0 (44)	1	396	67.9 (93)	1
Student, retired, sick-leave, parental leave or leave of absence, unemployed, other	66	20.0 (11)	1.31 (0.64–2.79)	172	32.1 (44)	1.13 (0.74–1.71)
**Partner**’**s employment status**						
Employed	262	73.5 (36)	1	414	78.9 (90)	1
Student, retired, sick-leave, parental leave or leave of absence, unemployed, other	121	26.5 (13)	0.78 (0.40–1.53)	111	21.1 (24)	0.99 (0.60–1.65)
**Children living at home**						
No	206	52.7 (29)	1	297	46.0 (63)	1
Yes	190	47.3 (26)	0.98 (0.55–1.73)	270	54.0 (74)	1.36 (0.93–2.01)
**Access to social support**						
Good	251	44.6 (25)	1	361	47.4 (64)	1
Poor/moderate	139	55.4 (31)	2.65 (1.49–4.71)	199	52.6 (71)	2.59 (1.74–3.85)
**Grown up in a home with violence**						
No	357	74.5 (41)	1	510	82.6 (109)	1
Yes/Unsure	38	25.5 (14)	4.37 (2.09–9.11)	55	17. 4(23)	3.09 (1.70–5.60)

Total sample n = 972.

In the adjusted multivariate analyses for psychological and physical/sexual IPV, age for men and partner’s country of birth for women no longer remained statistically significant (Table 5). Being single, widowed or divorced, having poor/moderate access to social support and having grown up in a home with violence remained associated with exposure to psychological and physical/sexual IPV. Moreover, having a lower educational level decreased the likelihood of reporting psychological IPV for both women (OR 0.48; 95% CI 0.27–0.83) and men (OR 0.45; 95% CI 0.22–0.92). Entering the excluded variables one at a time showed no significant contribution to the final models and no significant interactions were found (analyses not shown).

**Table 5:** **Adjusted associations between socio**-**demographic and psychosocial factors and exposure to psychological and physical**/**sexual violence**

	**Psychological violence**		**Physical/sexual violence**	
	**Men: N = 399**	**Women: N = 573**	**Men: N = 399**	**Women: N = 573**
**Civil status**				
(Married/cohabitant/registered partnership/boy- or girlfriend vs. Single/widowed/divorced)	3.25 (1.68–6.28)	3.92 (2.32–6.63)	2.67 (1.23–5.83)	3.33 (1.92–5.76)
**Access to social support**				
(Good vs. Poor/Moderate)	2.03 (1.27–3.25)	2.12 (1.44–3.12)	2.62 (1.42–4.83)	2.52 (1.66–3.84)
**Growing up in a home where violence occurred**				
(No vs. Yes/Unsure)	2.47 (1.19–5.13)	3.09 (1.62–5.88)	4.03 (1.84–8.83)	2.15 (1.18–4.14)
**Educational level**				
(Univ./High school vs. Compulsory)	0.45 (0.22–0.92)	0.48 (0.27–0.83)	-	-
**Age groups**				
(30–65 vs. 18–29)	1.60 (0.85–3.01)	-	1.96 (0.91–4.21)	-
**Partner**’**s country of birth**				
(Sweden vs. outside Sweden)	-	-	-	1.46 (0.82–2.60)

Total sample n = 972.

### Social consequences of violence on everyday life, own use of violence and reasons for using such violence

In total, 10.1% (n = 58) of the women and 6.5% (n = 26) of the men experienced social consequences of having been exposed to IPV (Table 6). Only women reported consequences related to children, such as taking the children away from the home (20.7% women vs. 0% men), whereas men more frequently reported working more than usual to keep away from home (34.6% men vs. 22.4% women). The differences between women’s and men’s responses were not statistically significant (p > 0.05).

**Table 6:** **Social and behavioural consequences of IPV and reasons for using violence**, % (**N**)

Number of respondents reporting social and behavioural consequences of IPV*	**Men N = 26**	**Women N = 58**
Work more than usual to keep away from home	34.6 (9)	22.4 (13)
Keep distance to common friends	15.4 (4)	24.1 (14)
Take the children away from the home	0 (0)	20.7 (12)
Change work	15.4 (4)	6.9 (4)
Avoid being at home when the children or no one else was at home	0 (0)	6.9 (4)
Move away from home	26.9 (7)	41.4 (24)
Divorce	15.4 (4)	34.5 (20)
Live under a secret identity	3.8 (1)	1.7 (1)
Number of respondents reporting reasons for using violence*	**Men N = 32**	**Women N = 55**
I felt afraid and did it in self-defence in a violent situation	18.8 (6)	30.9 (17)
I felt offended and/or hurt (saddened)	50.0 (16)	54.5 (30)
I did it in a quarrel since my partner or we both had used alcohol or drugs	31.3 (10)	16.4 (9)
I lost control	25.0 (8)	18.2 (10)

*Percentage is calculated based on those who responded to the question.

On the question of whether the respondent ever had used violence toward their intimate partner, 4.0% (n = 23) of the women and 4.3% (n = 17) of the men of the total sample answered in the affirmative. Out of these, the majority of women (82.6%; n = 19) and men (82.4%; n = 14) reported having both used and been exposed to IPV at some point in their lives. Finally, 2.1% women and 2.0% men had used physical violence, 1.4% women and 1.8% men psychological violence and one woman had used sexual violence towards a male intimate partner (not in Table).

Reasons for using IPV were given by 9.6% (n = 55) of the women and 8.0% (n = 32) of the men in the total sample. The most common reason was having felt offended and/or hurt (54.5% of the women and 50.0% of the men; Table 6). Other commonly reported reasons for using IPV included fear and self-defence (30.9% of the women and 18.8% of the men), alcohol or drug use (16.4% of the women and 31.3% of the men) and loss of control (18.2% of the women and 25.0% of the men). No statistically significant differences between women’s and men’s responses were observed.

## Discussion

Although past-year exposure rates to psychological, physical and sexual IPV were similar among women and men, earlier-in-life exposure to all three forms of violence was significantly higher among women. Psychological, physical and sexual IPV often co-occurred. Factors associated with all forms of IPV for both women and men were poor/moderate social support, having grown up in a home with violence and being single, divorced or widowed. There was a tendency for women and men to report different social consequences of IPV; however, these differences were not statistically significant.

Sweden is often considered to be one of the most gender equal countries worldwide with its high participation of both women and men in, for example, the labour force; decision-making posts, such as the Parliament; and higher education. There is generally a low tolerance of violence and using violence in an intimate relationship is punishable by law. There exist few studies that assess IPV in the general population of both women and men; the present findings confirm that IPV is common in Sweden and that continued efforts towards ending IPV are warranted. Professionals working within the health care sector should be sensitive towards the possibility that a male or female patient may have been exposed to violence in their intimate relationship and that this violence may take place in many different contexts and lead to a diverse set of consequences. Health professionals are in a unique and valuable position to detect and document IPV.

### Prevalence, frequency and co-occurrence of psychological, physical and sexual IPV

Previous studies have also found that past-year IPV estimates are similar in women and men, but that earlier-in-life IPV is higher in women [6,34,35]. It may be hypothesized that the two different time frames in part reflect differences in severity and impact of IPV between women and men, which have been found by previous studies [13,22]. For example, considering that men generally experience less threatening and severe forms of IPV, they may not consider it particularly salient to remember later in life. Similarly, given that women are generally exposed to more severe forms of IPV with higher levels of physical injury, coercive control and fear, they may be more likely to report such violence also later in life [36]. Given that several studies rely on past-year estimates–which, for example, have been considered to be the norm in community-based samples [37]–this finding suggests that gender differences in exposure to IPV might go unnoticed if earlier-in-life estimates are not accounted for.

Our past-year exposure rates are similar to those reported in previous studies, with the exception of sexual IPV, which is usually found to be more prevalent among women than men [34,38]. The earlier-in-life rates were somewhat lower in the current study compared to previous ones, [34,39] which may be due to actual differences in prevalence and to differences in the definitions of IPV. In line with previous population-based studies, [40] severe acts of IPV were more seldom reported and our sample consisted mostly of the comparatively less severe acts of IPV.

The finding that women were exposed to all three forms of IPV more often than men within the earlier-in-life time frame (22.1% vs. 11.5%) is supported by other studies [24]. However, corresponding rates during the past year were similar between women and men (4.5% vs. 3.9%). This further highlights the importance of considering the earlier-in-life time frame when assessing gender differences in exposure to IPV. Our exposure rates for psychological violence are higher than the other forms of IPV, which is in line with previous studies [4,24].

Finally, it is worth noting that while support has been found for the VAWI’s validity among the female sample used in the current study, [29] the VAWI’s conceptual model was only partially replicated among the male sample [28]. Instead, the boundaries between psychological, physical and sexual acts of violence were indistinct. Although further studies are needed in order to understand these results more fully, it nevertheless introduces a degree of uncertainty to our direct comparisons between women’s and men’s responses. Even if similar acts of violence are reported, the underlying constructs may differ. However, support for the instrument’s reliability was found among both samples.

### Associated socio-demographic and psychosocial factors

We found that both poor to moderate social support, having grown up in a home with violence and being single, widowed or divorced increased the likelihood of reporting exposure to psychological and physical/sexual IPV for both women and men residing in Sweden, which is in line with previous literature from other countries [24,41]. A possible explanation for the association between IPV and being single, widowed or divorced may be that the IPV was committed by a previous partner, which may be easier to report than violence experienced by a current partner.

Although most population-based studies investigate associated factors with physical and/or sexual IPV, our findings suggest that such factors are also associated with exposure to psychological IPV.

Educational level was also found to be associated with IPV, specifically having a university degree and/or having completed high school increased the likelihood for reporting psychological IPV for both men and women. Another population-based study assessing violence against men by several perpetrators and which was also conducted in Sweden has reported similar findings; [42] however, additional studies are needed to investigate this association further.

### Social consequences of violence to everyday life, own use of violence and reasons for using such violence

#### ***Social consequences***

Researchers have long underlined the importance of assessing consequences of IPV alongside the violent acts themselves as a way to consider the impact of IPV on women’s and men’s lives [35]. In the current study, a traditional gender structure emerged to some extent from the results: only women reported consequences which related to children, whereas men more often reported consequences related to work. As more women than men reacted by divorcing their spouse or moving away from home to protect themselves from IPV, a possible interpretation is that women felt more threatened by the violence and therefore took more measures to end the relationship than men. This finding is supported by a study where women were more likely to dissolve a heterosexual relationship than men if there occurred severe forms of violence, whereas women and men were equally likely to dissolve a relationship if there occurred less severe forms of IPV [43].

#### ***Own use of violence***

Although only 4.0% women and 4.3% men in the total population sample reported having used IPV sometime in their lives when assessed by a single item, a larger number of the respondents (9.6% women and 8.0% men of the total sample), however, gave a reason for having used IPV. This discrepancy may not be surprising as respondents may find it easier to give reasons for using violence than to define their use of violence as IPV, which has indeed been found to under-estimate true prevalence rates [44]. Both women and men under-report their use of IPV although some studies have found women to under-report to a lesser extent than men [18]. Reasons for this may be that women are more prone to remember their use of violence as they are transgressing a gender norm in using violence, whereas the use of violence is a more acceptable part of masculinity constructions and therefore may go more unnoticed or become normalized [11]. Our finding that some report own use of violence may suggest that IPV takes place in several contexts, including relationships where the respondent is both exposed to and exposes his/her partner to violence, as shown by previous studies [19,45].

#### ***Reasons given for using violence***

Reasons given for perpetrating violent acts may also reflect on the context in which the IPV took place [35]. The most commonly self-reported reason for using IPV by both the women and men was having felt offended and/or hurt, which has also been reported in the literature [45]. The second most reported reason by women was feeling afraid and using violence in self-defence in a violent situation. Previous studies have often found women to use IPV in self-defence [46]. The second most reported reason by men for having used violence was in a quarrel in which alcohol or illicit drugs was involved.

### Methodological considerations

The main known limitation of postal surveys is low response rates [47]. The current study included two reminders in an effort to minimize drop-out rates; however, the overall non-response rates were high for both women (38%) and especially for men (54.6%), which suggests caution in the interpretation of the results. The differing response rates among women and men also complicate the comparability of IPV between these two groups. Moreover, the response rates were lower among young, unmarried respondents, respondents with a lower annual income and respondents born outside Sweden. These groups have, especially among women [3], but also among men [48], been identified in the literature as particularly vulnerable to IPV. It is therefore possible that the current study has under-estimated the prevalence of IPV and consequently under-estimated the strengths of the associations between IPV and the socio-demographic factors. Also, an aggregate life-time variable was used to increase statistical power in the analyses assessing the associations between IPV and the socio-demographic factors. However, the exposure rates of IPV differed for women and men according to past-year and earlier-in-life time frames and it is therefore possible that the associated factors would have differed for women and men had they been assessed separately for the two time-frames. Finally, as the study was cross-sectional, we are not able to draw any conclusions about cause and effect.

Furthermore, both women and men have been found to under-report their exposure to violence in heterosexual intimate partnerships, [18] although some studies have found that men may also over-report such exposure while at the same time being perpetrators of IPV [49]. Moreover, studies have found low to moderate inter-spousal agreement on the occurrence of IPV among heterosexual couples, both with regards to the frequency and the severity of the violence [18]. Additionally, the violent acts that the respondents have been exposed to may differ in context. As such, we are not able to differentiate between, for example, a shove made in self-defence and a shove made in a context of assault and intimidation [50]. As in all survey-based results relying on self-reports, caution should be used in interpreting the results.

Constructions of masculinity and femininity may influence how a person makes sense of and reports IPV, which in turn shapes results and conclusions [8,11,18]. An important research question is thus whether men and women define and report IPV in different, gendered ways. Future studies on this topic would conceivably help in interpreting survey-based data.

## Conclusions

The findings confirm that exposure to violent acts by an intimate partner is common in Sweden; however, the high non-response rates, especially among men, calls for careful interpretation of the results. Although past-year IPV exposure rates were similar in women and men, earlier-in-life exposure rates were higher in women. This suggests that the earlier-in-life time frame is important when assessing gender differences of IPV and future studies should consider it alongside past-year prevalence. Further in-depth, qualitative studies that probe deeper into the context of IPV in ways that consider masculinities, femininities, power and gender orders would contribute to a deeper understanding of the gendered matter of IPV.

## Competing interests

The authors declare that they have no competing interests.

## Authors’ contributions

LN conducted all analyses, wrote the first draft of the manuscript and rewrote new drafts based on input from co-authors. CT and VE discussed and gave input on manuscript drafts. GK designed the project, planned the analyses and gave input on manuscript drafts. All authors read and approved the final manuscript.

## Pre-publication history

The pre-publication history for this paper can be accessed here:

http://www.biomedcentral.com/1471-2458/13/845/prepub
